# Exploring novice nurses’ needs regarding their work-related health: a qualitative study

**DOI:** 10.1007/s00420-015-1022-5

**Published:** 2015-01-30

**Authors:** Sarah M. Ketelaar, Karen Nieuwenhuijsen, Monique H. W. Frings-Dresen, Judith K. Sluiter

**Affiliations:** Academic Medical Center, Coronel Institute of Occupational Health, University of Amsterdam, PO Box 22700, 1100 DE Amsterdam, The Netherlands

**Keywords:** Health personnel, Needs assessment, Nursing students, Occupational health services, Primary prevention

## Abstract

**Purpose:**

To investigate Dutch novice nurses’ experiences and needs regarding occupational health support to prevent work-related health problems and to keep them well-functioning.

**Methods:**

A qualitative interview study was conducted with six nursing students and eight newly qualified nurses. The interviews covered three topics: experiences with the link between work and health, received occupational health support, and occupational health support needs. Data were analysed using a grounded theory approach.

**Results:**

Participants reported experiences with work-related health problems early in their career and described experiences with how health problems lead to suboptimal work functioning. Occupational health support needs included knowledge and psychosocial support during nursing education, e.g. through paying attention to dealing with shift work, or career counselling. Also, they reported a need for knowledge and psychosocial support at the start of their clinical placement or new job in the hospital, e.g. information from occupational health services or having a mentor. Furthermore, they reported that occupational health support requires a more general place at work through offering knowledge, e.g. tailored advice on proper lifting position; psychosocial support, e.g. positive team atmosphere; and physical support, e.g. suitable preventive measures.

**Conclusions:**

Occupational health support for novice nurses is important, since they already experience work-related health problems and suboptimal work functioning due to health problems early in their career and while still in training to be a nurse. Novice nurses should be given more knowledge and support to help them stay healthy and well-functioning in their work. This is a joint responsibility of nurse educators, the employer and occupational health services.

## Introduction

Due to their nature, some jobs entail job demands which cannot be eliminated or adapted to make them less unhealthy. Nursing forms one of these jobs. Working as a hospital nurse entails several work-related physical and psychosocial health risk factors including some that cannot be avoided completely, such as lifting patients, working with infectious patients and their body fluids, shift working, and dealing with workplace aggression. Nurses’ job demands may lead to health problems (Sluiter et al. 2003). Various studies have found a high prevalence of certain health problems in nurses, such as low back pain (d’Errico et al. 2013), skin problems (Cherry et al. 2000) and mental health problems (Gao et al. 2012).

Nursing students as well as newly qualified nurses who have only just recently started working as a qualified nurse seem particularly vulnerable to developing health problems and making mistakes, precisely because they are inexperienced and therefore do not have fully developed skills yet. Additionally, they are more vulnerable because they have difficulty asking for help (Evans 2001). Research has indeed shown a high prevalence of musculoskeletal problems (Cheung 2010; Smith and Leggat 2004) and burnout (Rudman and Gustavsson 2011, 2012) in novice nurses and a decline in self-reported health between the last study year and the following 3 years (Hasson et al. 2010). Also, nursing students are at risk of experiencing needlestick injuries and other forms of exposure to biological material (Petrucci et al. 2009; Small et al. 2011). This alone forms an incentive to pay attention to nurses’ health, but health problems in nurses are also known to have effects on their work functioning, with possible consequences for patient care (Gärtner et al. 2012; Letvak et al. 2011, 2012).

All in all, it seems important to offer novice nurses occupational health support, defined as support to prevent them from developing work-related health problems and to help them to function optimally. To our knowledge, no research has focused on this kind of support for novice nurses. Therefore, in this study, we investigated the following topics in novice nurses: (1) their experiences with the link between their work and their health; (2) the occupational health support that they already receive; and (3) their further needs for occupational health support.

## Methods

### Context

In the Netherlands, educational institutes are required to offer their students a safe work environment. In addition, the Dutch Working Conditions Act (2007) states that employers are required to pursue an occupational health and safety policy targeted towards optimal occupational health and safety. This Act also applies to students working in clinical placements.

### Participants

We aimed to interview novice nurses with at least some experience in clinical practice, so that they would be able to reflect on occupational health support. This population was operationalised as (1) third or fourth year regular students, or workplace learning students, both in higher education and (2) newly qualified nurses currently working as a nurse and with a maximum of 5 years of working experience after completing their studies.

Participants were recruited using methods such as notices on websites and social media, posters and flyers. We initially aimed to recruit 12 participants (three students and three newly qualified nurses in general healthcare and three students and three newly qualified nurses in mental healthcare) and to continue recruiting participants until no new information was given. To refine the interviewing method, another two interviews were conducted. The Medical Ethics Committee of the Academic Medical Center in Amsterdam, the Netherlands, approved this study.

### Data collection

The interviews were conducted face to face by the first author (S.K.) at a time and place which suited the participant. The interviews were audio-recorded and typically lasted 1 h. Written informed consent was obtained from all participants. Three topics were covered during the interview using semi-structured open-ended questions (Box 1):

**Box 1:** Topics and subtopics of the interview

Experiences with the link between work and health (to introduce the main topic of the interview):
The impact of their work on their health (to elicit experiences, several short case descriptions were prepared and given to the participant to read during the interview when useful)
The impact of health problems on work functioning
Occupational health support that they received to:
Prevent them from developing work-related health problems
Help them to function optimally in spite of health problems
Occupational health support needs to:
Prevent them from developing work-related health problems
Help them to function optimally in spite of health problems

1. Experiences with the link between work and health.
2. Received occupational health support.
3. Needs for occupational health support.

After each interview, the first author (S.K.) wrote a short summary and sent this to the participant to check whether reported needs had been interpreted correctly.

### Data analysis

All audio-recorded interviews were transcribed verbatim by the first author (S.K.). Using the software program MaxQDA version 11, the transcripts were analysed using a grounded theory approach, identifying themes that emerged from the data (Pope et al. 2000). We alternated between coding earlier interviews and conducting additional interviews to allow for further exploration of new subthemes in subsequent interviews and to assess data saturation. Towards the last interviews, refinements of the thematic structure were made rather than new categories, indicating data saturation.

During the analysis process, all transcripts were open-coded to identify all important aspects that provided an answer to the research question. Two-thirds of the transcripts were open-coded by both the first (S.K.) and second author (K.N.), while the remainder was open-coded by the first author (S.K.) only and checked by the second author (K.N.). In the second phase of analysis, relationships between open codes were sought and (sub)categories were formed through constant comparison. In the third and final phase of analysis, main themes were formed that answered the research question. The themes and (sub)categories were discussed among all authors to increase reliability of the analysis. The code system is available on request from the second author (K.N.).

## Results

### Participant characteristics

Six nursing students and eight newly qualified nurses, all females, applied to participate. Participating nursing students were aged 23–45 (mean = 31, SD = 8.3), while participating newly qualified nurses were aged 23–40 (mean = 29, SD = 6.1). Eleven participants studied or worked in general healthcare (mainly hospitals), two studied or worked in mental healthcare, and one worked in homecare. Working experience of newly qualified nurses after completing their studies ranged from 5 months to 5 years.

### Experiences with the link between work and health

#### Work influencing health

Most participants had experiences with work-related health problems, such as musculoskeletal problems; stress and worrying; anxiety; fatigue; sleeping problems; and infectious diseases such as influenza. Reported aspects of working nursing causing these health problems were physical work demands such as lifting patients and standing or walking all day; disagreeable contact with patients, patients’ family or colleagues; having to do a lot of work in a limited time; accidents with biological material and otherwise being exposed to infectious agents; working shifts; or making mistakes. It is notable that some participants believed that problems such as back pain are just part of the deal when working in nursing:

Everyone knows, there’s all this equipment that you can use, and everyone knows that it’s heavy work, it’s kind of part of the job. You know, you have all your equipment, but if a patient makes an unexpected movement you can’t do anything but step in, and you know, it puts a strain.., it’s very physical work. (ID2)

#### Health influencing work functioning

Health problems were reported to lead to several forms of suboptimal work functioning, such as being unable to perform physical tasks; impaired contact with patients; lack of concentration; forgetting things and making mistakes; and being unable to respond quickly when faced with unexpected events.

### Received occupational health support

In Box 2, the forms of occupational health support are presented that participants reported to have experienced. As a general form of support, some participants stated that they had received information at the start of their career from the occupational health services department, telling them where they could get help if they experienced problems. Other forms of support that participants received for either prevention of work-related health problems or for staying well-functioning in spite of health problems are described below.

**Box 2:** Received occupational health support

Prevention of work-related health problems
Peer support
Advice from professionals
Being able to ask colleagues or supervisor for help
Existing measures to deal with incidents (e.g. protocols)
Preventive measures (e.g. personal protective equipment and ergonomic devices)
Lessons in nursing education (learning, practicing, and sharing experiences)
On-the-job training (e.g. clinical lessons)
(Psychosocial) support from school
Distribution of workload
Knowing and indicating your personal limits
Support to stay well-functioning in spite of health problems
Peer support
Being assigned different or adapted tasks
Working less hours or taking more breaks
Knowing and indicating your personal limits
Being able to ask colleagues or supervisor for help
General support
Introductory information from occupational health services

#### Prevention of work-related health problems

Peer support was often mentioned as important to be able to cope with the job demands. This peer support ranged from giving each other tips about bringing the patient’s bed at the right height to facilitate lifting the patient, to giving support when dealing with an emotionally demanding event. Nevertheless, when the novice nurse makes a mistake, it can be difficult to talk about to colleagues, although others might have had similar experiences:

I think I sometimes keep things to myself too much. I should talk to colleagues about it more, because I constantly have this idea that I’m always the one who makes mistakes. But the more you talk to colleagues about it, you hear, oh, yes, I had that once too, and then you find out that other people also make mistakes and that you are not the only one. (ID9)

Participants also reported receiving advice from professionals. For example, they reported receiving advice from occupational health and safety professionals and the hygiene and infection control department regarding preventive measures when working with infectious patients and their body fluids. Participants also reported receiving advice from a physiotherapist, for instance about how to improve their lifting position or about which shoes are most suitable to wear at work. Several participants mentioned that they have a colleague who also functions as an ergonomic coach in the workplace. Experiences with this ergonomic coach varied, as some participants reported receiving advice from this coach but others stated that they heard too little from him or her.

Asking colleagues or their supervisor for help to deal with situations which could impact their own health was another form of support that participants received. However, asking for help can be hard because it may give the impression that the student or newly qualified nurse is not sufficiently capable. Other reasons not to ask for help were, for instance, feeling ashamed or not having the chance or time to receive help.

Participants reported existing measures to deal with incidents such as needlestick injuries or incidents of aggression as a form of occupational health support, although some participants noted that protocols on how to deal with patients with infectious diseases are not always clear. In addition, existing preventive measures were experienced as a form of support, such as personal protective equipment and equipment for lifting patients. However, time restraints or not knowing how to use equipment sometimes caused participants not to apply these measures. One student also mentioned that her dependent position sometimes made it hard to apply preventive measures if her colleagues showed no intention of using them:

Especially the slightly older nurses, I noticed, who are so used to doing it a certain way, they do it that way, and sometimes it can be difficult as a student to say yes but I want the bed to be at working height, or, you know, wait until I am completely ready for it. That can sometimes be difficult. You don’t want to… (…) They have been doing it for so long, and you have just started, and then you start complaining, you know, that’s a bit… (ID1)

Additionally, wearing personal protective equipment is sometimes experienced as too warm and uncomfortable.

Participants also received support through classes during nursing education in which information was given, experiences could be shared, or lifting positions could be practiced. However, it was also stated that the information and opportunity to practice given in class were limited. Participants also reported receiving some education at work regarding lifting positions, safety measures and dealing with incidents of aggression, mostly given in so-called clinical lessons.

During their studies, participants received support from their nurse educators and fellow students, although it was also stated that when students reported that the work was too much or that they were very tired, the teachers replied that this was part of the job and they just had to get used to it. Another participant mentioned that the people from her educational institute who could offer support were mostly hard to reach.

Additionally, participants reported that the workload is distributed between colleagues, and many participants stated that it is important to know and indicate your own limits and boundaries to prevent yourself from developing health problems.

#### Support to stay well-functioning in spite of health problems

Peer support was reported as a form of support to stay well-functioning in spite of health problems, mainly through sympathising or through taking their health problems into account. Another important form of support that many participants mentioned was the possibility of performing different or adapted tasks when suffering from health problems. This varied from performing completely different tasks such as administrative work or processing patient admissions to only taking care of a specific patient population, e.g. only patients with less need for care, or no heavy patients. One participant who had lost her father not long before said:

A few months ago I didn’t need to take care of patients who were dying, I said it still really troubles me, it really makes me think of my father for example, then I really didn’t have to do it. That’s very much taken into account. (ID10)

The possibility of working less hours per day or taking more breaks was also mentioned, although this is not always put into practice easily. In any case, it is important to know and indicate your own limits and boundaries. Many participants indicated that when experiencing health problems they asked for help, mostly from their supervisor. Nevertheless, the dependent position as a student was reported once more as an impeding factor to ask for help.

### Further occupational health support needs

The further occupational health support needs that participants reported are presented in Box 3, and further elaborated on below.

**Box 3:** Further occupational health needs

Proper support as a novice nurse
Preparation for clinical placements
Introduction day
Being an ‘extra’ in the first period
Introductory information from occupational health services on nurses’ own health and work
Encouragement to reflect on own coping
Regular evaluation meetings with supervisor
Having a mentor
Paying attention to first experiences (e.g. patient dying, working with wounds)
Training in how to cope with the work
Knowledge
Dealing with needlestick injuries
Dealing with the risk of infection
Existing preventive measures and health promotion
How to cope with rotating shifts
Proper footwear
How to cope with physical job demands
Where to ask for help when needed
Tailored advice on work posture and lifting patients
Psychosocial support
Counselling
Feeling free to ask for help
Discussing unacceptable behaviour of others
A periodical discussion about how things are going
Paying attention to the team atmosphere
Taking into account personal work schedule preferences
Finding a job that fits your preferences
Physical support
Proper equipment and personal protective equipment
Support to keep a healthy lifestyle
On-the-job massages

#### Need for proper support as a novice nurse

It was stated that during education, students should be prepared for their clinical placements. Also, an introduction day for new employees was mentioned as beneficiary. A guided tour of the hospital and important departments should be part of this introduction. In addition, being an extra employee in for example the first 2 months which enables new employees to watch and run along with colleagues and settle into the workplace is important.

It was indicated that the health check at the start of their placement or career would be a good opportunity for the occupational health services to give novice nurses advice about their own health, how to stay healthy and where to ask for help if needed and to check if they would like to talk about any health problems.

Students should also be encouraged to think about how they can protect themselves from developing work-related health problems and what their way of coping is and should be. In addition, a meeting of the new employee with the supervisor after 2 weeks should be incorporated, in which the new employee can indicate what he or she needs and how he or she can be supported by the team or the hospital. Furthermore, it was mentioned that fixed moments for evaluation should be planned, starting from 1 month after starting the work, to talk about things such as how do you find your new job and what problems have you encountered. In addition, new employees should have a mentor, for instance a senior nurse who is not their supervisor, whom the new employee can turn to with any problems that they encounter:

When interns from for instance psychology or medicine come here, they are taken under someone’s wing. A whole system has been thought out for them. They get a mentor and it is thought out who they can watch and run along with. But when you start working here as a nurse, you basically start working right away. You are supervised, but that purely regards the content of the work. So it would be good if someone is appointed for nurses as well, to whom you can turn, maybe for the first year or so, to ask how things are done here or how do you deal with that, or… Not only regarding content of the work, but also, well, a sort of mentor. (ID13)

It was also mentioned that sometimes colleagues are not sufficiently attentive that nursing students might not have experienced certain emotionally demanding events, e.g. a patient dying. More attention should be given by their supervisor, asking them whether they have experienced this before and how they feel about it. The same holds for experiencing ‘new things’: it was regarded beneficiary if students are given the opportunity to build up their tolerance at their own pace:

Just that you have the possibility to indicate how you feel and that it is not weird that you have not seen many open wounds and seen a lot of blood or experienced that smell (…). That you know you can take your distance for a minute and that you are allowed to build up your tolerance. (…) I noticed that I thought it was nice to know [that you were given that possibility], because I thought, it’s really weird if I suddenly walk away (…). That’s another one of those intern things, you feel that you have to be able to cope with everything, because after all, you chose to go into nursing so you have to be able to see everything. (ID9)

It was also felt that new employees should receive a group training in how to cope with the work and how to detect early signs of mental health problems and that they should be encouraged to ask for help early to prevent development of mental health problems.

#### Need for knowledge

Participants reported not knowing what to do or where to go when sustaining a needlestick injury. Also, participants reported wanting to know after sustaining a needlestick injury when they would hear what the results of their blood sample was, how likely it was that they had contracted something, and what signs they should be attentive of after the incident.

Regarding infectious diseases, some participants reported that regulations on dealing with these diseases are unclear and sometimes differ per professional whom is asked for advice. A need was reported for knowledge on how to use personal protective equipment correctly and information regarding the risks of working with an infectious patient for nurses themselves. Also, participants wanted more general information on reasons for patients needing to go into isolation, what you should pay attention to, and why you should use what personal protective equipment.

Participants also stated that they would like information about the effect that working rotating shifts can have on your body, as well as tips about how to deal with rotating shifts:

From one of the professional associations for nurses I got advice for during your night shift, what to eat and what not to eat. And I notice that such tips really do me good. (…) I experience that as positive, and I immediately put them into practice, such as I shouldn’t take protein because it makes you really tired (…), but I should eat fruit and light things because you can tolerate those well. Such tips. (ID12)

Another topic that participants reported wanting more information about, was how to cope with physical job demands. How can musculoskeletal problems be prevented; what is the best work posture for which task; what equipment can be used; and where can you find this equipment? Several participants also reported a need for tailored advice on how to lift a patient. They stated that they wanted a professional to observe them while lifting or shifting a patient and then give them personal advice on how their posture could be improved to prevent musculoskeletal problems.

#### Need for psychosocial support

The need for counselling regarded a form of periodic peer counselling either with fellow students or with colleagues, in which participants can discuss any problems they had encountered. Individual counselling was also mentioned by some participants as something they would like to see being set up. Someone also mentioned that one colleague could have the specific function to offer counselling after experiencing emotionally demanding events. For students, offering the possibly to talk to a psychologist or social worker could be a good solution or matching a student with a colleague in the clinical placement to talk to when needed.

Also, participants felt that it should be made easier to ask for help. Employers could play a part in this:

I think it would be good to get a bit more information about [the link between your work and your health], as an employee. And that it’s not strange if you mention it sometimes, because now it’s usually perceived as complaining, or, (…) you feel a bit uncomfortable if you say something about it. While I think it’s good to break through that a little, to make it possible to just talk about it. (…) And that you get the idea that it’s better understood, or that it’s not strange if you experience [problems]. (ID5)

Additionally, the occupational health services could be more open for employees to ask for help anonymously, for instance through an open consultation hour:

It might be a good idea for the occupational health services to provide some kind of open consultation hours, or just times that you can walk in with things and say, I don’t feel well and I want to talk about it. I don’t think we have that now. Not that I know of anyway. (ID5)

For students, asking for help can be difficult because of their dependent position. Colleagues can help lower the barrier:

That they tell you, you can make mistakes here, it’s for your own safety to just tell us everything that troubles you, and don’t try to solve it yourself but just realise that you are a student, (…), and that you feel safe in the team. That people can really help you and that you need to be open because it’s better for everyone’s safety. (ID2)

Throughout the study, participants made it clear that team atmosphere is very important for employees’ health and well-functioning. Some participants further described that they felt more attention should be paid to the team atmosphere and tackling on-the-job gossiping, for instance through team building activities.

Some participants stated that they would like their employer to take into account their personal situation and personal needs regarding their work schedule:

That you are a bit more flexible and when at a certain moment you say, I can’t handle the late shifts or the night shifts so well anymore or, I prefer day shifts, that that’s possible. You know, even just the rule that you have to solve it yourself, but that that is allowed. [Swapping shifts with a colleague] is allowed, but only to a certain extent, and it’s so limited that there are very little opportunities to swap shifts. (ID11)

Participants also reported how important it is to find a job that matches you personally. During their studies, students could be encouraged to reflect on what suits their preferences, using a form of career counselling.

#### Need for physical support

Regarding proper equipment and personal protective equipment, it was mentioned that they would like their organisation to reimburse proper shoes and to offer appropriate equipment for lifting and shifting patients. Regarding support to keep a healthy lifestyle, participants stated that they would like their employers to offer a discount on gym membership and to pay attention to healthy foods, for instance by offering free fruit during lunch hours. Another participant stated she would like some time to eat properly, since her work is so tightly scheduled that she does not have time to eat, let alone eat healthy.

#### Ideas for implementation

Participants provided various ideas on how occupational health support might be given to them. Information about possible effects on nurses’ own health should be integrated in their education. For instance, when learning about handling needles during nursing education, the implications of sustaining a needlestick injury could also be discussed. At the beginning of the placement or career, general information about the most frequently encountered work-related health topics at that specific workplace and the occupational health and safety rules could also be given.

Regarding dissemination of information on occupational health topics, participants reported several methods that they considered useful. Information could for example be given in class during nursing education, in clinical lessons at work, through watching and learning from colleagues in the beginning of their placement or career, and by encouraging novice nurses to read the protocol. They also mentioned the possibility of information leaflets or posters at appropriate places and paying organisational attention to the subject, for instance by organising a special week about an important topic. In addition, conferences for nurses and the professional association could provide information on occupational health topics. Mostly, participants stated that repeating information once in a while would be beneficiary. This was especially the case for information about dealing with physical job demands.

The key elements of experienced and required occupational health support are provided in Box 4. The elements have been categorised into support that should be given during nursing education, support that should be given at the start of the clinical placement or new job and support that should be given a more general place at the workplace.

**Box 4:** Key elements of novice nurses’ occupational health support needs

During nursing education
Knowledge
Education on occupational health topics: dealing with needlestick injuries; dealing with infectious diseases; possible preventive measures and health promotion; coping with rotating shifts; proper footwear; how to cope with physical job demands and where to ask for help when needed
Psychosocial support
Preparation of students for clinical placements
Career counselling
Appropriate support from nurse educators
Peer support from fellow students
At the start of the clinical placement or new job
Knowledge
Introduction day including a tour of the hospital
Information from occupational health services at the start of their placement/career
Being an ‘extra’ in the first months
Psychosocial support
Discussion between new employee and supervisor about personal support needs
Reflection on coping strategy and how to protect themselves from developing work-related health problems
Mentor whom the new employee can turn to with any questions or problems
Proper support during or after emotionally demanding events, opportunity to build up tolerance at one’s own pace
Group training in how to cope with the work and how to detect early signs of mental health problems, including encouragement to ask for help early
At work in general
Knowledge
Education at work on occupational health topics: dealing with needlestick injuries; dealing with infectious patients/agents; possible preventive measures and health promotion; coping with rotating shifts; proper footwear; how to cope with physical job demands and where to ask for help when needed
Tailored advice on work posture and lifting patients
Occupational health advice from professionals
Psychosocial support
Peer support from colleagues
Attention for team atmosphere
Counselling, individual or through periodic peer counselling either with fellow students or with colleagues
Being able and feeling free to ask colleagues or supervisor for help
Possibility to ask occupational health services for help anonymously
Fixed evaluation moments
Knowing and being able to indicate one’s own limits and boundaries
Personal situation and personal needs regarding work schedule taken into account
Physical support
Measures/protocols to deal with incidents; discussing unacceptable behaviour
Preventive measures
Possibilities to distribute workload between colleagues
Possibilities to performing different or adapted tasks or working less hours per day when suffering from health problems
Support to keep a healthy lifestyle
On-the-job massages

## Discussion

The main aim of this study was to identify novice nurses’ experiences regarding support that they experience and their further support needs to prevent them from developing work-related health problems and to help them to function optimally in spite of health problems. Our findings indicated that occupational health support could be given during nursing education, at the start of their clinical placement or new job, and at work in general. Occupational health support needs during education covered knowledge (e.g. paying attention in class to dealing with shift work) and psychosocial support (e.g. career counselling). For the start of their clinical placement or new job, novice nurses also report a need for knowledge (e.g. information from occupational health services) and psychosocial support (e.g. having a mentor). In addition, at work in general, novice nurses report a need for knowledge (e.g. tailored advice on proper lifting position), psychosocial support (e.g. a positive team atmosphere) and physical support (e.g. suitable preventive measures).

### Interpretation of findings

The transition from being a nursing student to being a qualified nurse has received much attention in scientific literature (Jewell 2013; Phillips et al. 2014). However, literature on occupational health support to prevent novice nurses from developing work-related health problems and to help them function optimally in spite of any health problems was lacking. This study has provided knowledge on the novice nurses’ needs regarding occupational health support. We found that occupational health support should not only be given while on clinical placement or at work, but also during nursing education. The British Royal College of Psychiatrists (2011) also reflects that healthcare students’ help-seeking might be impeded due to fear of being suspended or excluded from the course, underlining the importance of providing help early in their career.

Furthermore, a place for occupational health support is required at the workplace. Our findings indicated that some aspects of occupational health support require extra attention at the start of a clinical placement or the new job. For example, a simple introduction to the workplace but also receiving information from the occupational health services. Psychosocial support at the start was also considered favourable, for instance by being paired to a mentor. Other studies underline the benefits of such support (Jewell 2013).

Nevertheless, ongoing occupational health support for nurses is also required. One important element of more general occupational health support addressed by our participants is a culture of support among team members, helping each other and treating each other with respect. The importance of a culture of support among team members is reflected by other studies, finding that a positive atmosphere leads to positive effects on nurses’ quality of life (Bourbonnais et al. 2004), nurses’ well-being (Utriainen et al. 2014), and proactive responding to mental health issues of colleagues (Moll 2014). A culture of support also appears to form a buffer for developing mental health problems for healthcare workers (Gao et al. 2012; de Jonge et al. 2008), and discussing details of medical errors with colleagues was found to be valuable for individual and team recovery (Sirriyeh et al. 2010).

Acquiring knowledge on certain topics intrinsic to the job of nursing was another reported need. For instance, our participating novice nurses’ reported that although certain personal protective equipment and ergonomic equipment is available in the workplace, many nurses do not know how to use this equipment properly. According to the Dutch Working Conditions Act (2007), the employer should give the employee sufficient and adequate instructions and training about among others the risks within the organisation and how to deal with these risks in a safe and healthy way, but it seems that this could be improved.

A need for knowledge on dealing with physical job demands was also reported by our participants. The time spent on education in ergonomics in the Dutch educational institutions offering healthcare studies is limited when considering the amount of physical work that nurses perform (de Vries et al. 2011). In the workplace, the inadequate knowledge and use of equipment that has been developed to deal with the physical as well as the mental job demands of healthcare workers is also recognised (Dekker et al. 2007; Douwes et al. 2008; Koppelaar et al. 2009). This underlines the importance of more and appropriate education regarding these aspects.

Working in mental healthcare generally poses less physical strain for nurses when compared to working in general healthcare. This was reflected in our findings that participating mental healthcare nurses had no experience with physical health problems due to their work and also did not report occupational health support needs regarding physical job demands. Therefore, the nursing specialisation should be taken into account when implementing occupational health support.

### Methodological considerations

We chose a qualitative research methodology, so that the topic could be inductively explored from the perspective of the novice nurses’ themselves. Furthermore, we chose to conduct individual interviews, to thereby enable going into the personal situation in detail. This proved a valuable approach, since the participating novice nurses had quite different stories and not all were as familiar with the topic of occupational health. In addition, conducting individual interviews prevented participants from experiencing peer pressure and therefore not reporting all experiences and needs (Tong et al. 2007).

We conducted interviews with both nursing students with clinical placement experience as well as newly qualified nurses who have already gone through the transition from student to qualified nurse. Incorporating these different perspectives has extended our knowledge on novice nurses’ needs along the spectrum from being a nursing student to having started working as a qualified nurse recently.

Although we have used an extensive recruitment strategy to attract respondents, we were unable to recruit male novice nurses. In the Netherlands, 16 % of workers in healthcare and welfare are male. Therefore, it would have been optimal to interview at least one or two male novice nurses, as perhaps they might have had a different perspective on occupational health support.

### Implications for practice

Our findings underline the importance of paying attention to occupational health support early, to prevent novice nurses from developing work-related health problems and to help them function optimally in spite of health problems.

Furthermore, our study indicated that novice nurses should be offered more knowledge and support regarding their occupational health. Nurse educators, employers who offer clinical placements for nursing students and who employ nurses, as well as occupational health services for healthcare employees each bear part of the responsibility to address these elements. An important starting point is for nurse educators to include a component in the curriculum offered to nursing students, teaching them about health risks due to working in nursing, consequences of these health risks and, most importantly, teaching them how to prevent these consequences. Occupational health services are advised to better acquaint nurses with available occupational healthcare at the start of their career. In addition, employers should take on their responsibility to take good care of their workers, and thus to provide them with sufficient knowledge and support to help them stay healthy and well-functioning in their work.
